# Genomic Epidemiology of *Salmonella* Infantis in Ecuador: From Poultry Farms to Human Infections

**DOI:** 10.3389/fvets.2020.547891

**Published:** 2020-09-29

**Authors:** Lorena Mejía, José Luis Medina, Rosa Bayas, Carolina Satan Salazar, Fernando Villavicencio, Sonia Zapata, Jorge Matheu, Jaap A. Wagenaar, Fernando González-Candelas, Christian Vinueza-Burgos

**Affiliations:** ^1^Instituto de Microbiología, Colegio de Ciencias Biológicas y Ambientales, Universidad San Francisco de Quito, Quito, Ecuador; ^2^Institute for Integrative Systems Biology, University of Valencia, Valencia, Spain; ^3^Unidad de Investigación de Enfermedades Transmitidas por Alimentos y Resistencia a los Antimicrobianos (UNIETAR), Facultad de Medicina Veterinaria, Universidad Central del Ecuador, Quito, Ecuador; ^4^Centro de Referencia Nacional de Resistencia a los Antimicrobianos, Instituto Nacional de Investigación en Salud Pública “Leopoldo Izquieta Pérez”, Quito, Ecuador; ^5^Department of Food Safety and Zoonoses, World Health Organization (WHO), Geneva, Switzerland; ^6^Department of Infectious Diseases and Immunology, Faculty of Veterinary Medicine, Utrecht University, Utrecht, Netherlands; ^7^Wageningen Bioveterinary Research (WBVR), Lelystad, Netherlands; ^8^Joint Research Unit “Infection and Public Health” FISABIO-University of Valencia, Valencia, Spain; ^9^CIBER (Centro de Investigación Biomédica en Red) in Epidemiology and Public Health, Valencia, Spain

**Keywords:** *Salmonella* Infantis, ST32, broiler, WGS, Ecuador, megaplasmid, multidrug resistance (MDR)

## Abstract

*Salmonella enterica* is one of the most important foodborne pathogens around the world. In the last years, *S*. *enterica* serovar Infantis has become an important emerging pathogen in many countries, often as multidrug resistant clones. To understand the importance of *S. enterica* in the broiler industry in Ecuador, we performed a study based on phenotypic and WGS data of isolates from poultry farms, chicken carcasses and humans. We showed a high prevalence of *S. enterica* in poultry farms (41.4%) and chicken carcasses (55.5%), but a low prevalence (1.98%) in human samples. *S*. Infantis was shown to be the most prevalent serovar with a 98.2, 97.8, and 50% in farms, foods, and humans, respectively, presenting multidrug resistant patterns. All sequenced *S*. Infantis isolates belonged to ST32. For the first time, a pESI-related megaplasmid was identified in Ecuadorian samples. This plasmid contains genes of antimicrobial resistance, virulence factors, and environmental stress tolerance. Genomic analysis showed a low divergence of *S*. Infantis strains in the three analyzed components. The results from this study provide important information about genetic elements that may help understand the molecular epidemiology of *S*. Infantis in Ecuador.

## Introduction

Foodborne infections caused by *Salmonella enterica* are of primary importance worldwide. The WHO estimates that *Salmonella* causes more than 153 million illnesses, 120,281 deaths, and 8.27 million disability-adjusted life years annually (1). As foodstuffs can be contaminated in several parts of the food chain, a “from farm to table” approach is necessary to understand the epidemiology of *Salmonella*. Although *Salmonella* can contaminate vegetables, food-producing animals, especially poultry, are considered important sources for human infections (2, 3). In Latin American countries, poultry is one of the main sources of protein of animal origin. This is the case of Ecuador, where poultry meat is the most consumed commodity with a yearly per capita consumption of 30.4 Kg (4).

Previous investigations in Latin America showed that *Salmonella enterica* serovar Infantis is an increasingly important serotype on poultry farms (5–9). Moreover, this serotype has also been reported to cause infections in local inhabitants and travelers that have visited Latin American countries (10–12). However, no genomic data considering isolates of *Salmonella* originated from animals, foodstuff, and humans have been released in Latin America so far.

Antimicrobials are commonly used in poultry production as both therapeutics and growth promoters. However, even when antimicrobials are used under technical criteria, they can select resistant strains of *Salmonella* that pose a public health problem when reaching consumers (13). This is of special concern in developing countries where the misuse of antimicrobials and lack of control is an issue to be addressed.

There is a wide diversity of virulence factors that are essential for pathogenicity of *Salmonella* in host cells. Among these factors, fimbriae, flagella, plasmids, pathogenicity islands, toxins, and secretion systems are the more frequently associated to pathogenic strains of *Salmonella* (14).

This research was aimed at describing by phenotyping methods and whole-genome sequencing, the antimicrobial resistance (AMR) characteristics and genetic profiles of *Salmonella* isolates obtained from broiler farms, broiler carcasses and humans in Quito—Ecuador.

## Materials and Methods

### Study Design and Sampling

Samples for *Salmonella* isolation were collected weekly from November 2017 to November 2018 following the guidelines of the document: “Integrated Surveillance of Antimicrobial Resistance in Foodborne Bacteria” by the World Health Organization (15).

Poultry Farms: 133 flocks from 69 farms were sampled during the study period. For every sampled flock, 25 caeca from 25 chicken were randomly collected at the slaughterhouse level and transported to the laboratory in an ice-box within the next 2 h. Caecal samples are recommended by the WHO because they provide a higher recovery of isolates and better represent contamination of individual animals at the farm level (15). At the laboratory, a sample pool of 25 g was obtained for bacteriological isolation as previously described (8).

Chicken carcasses: 335 carcasses were collected in three kinds of markets as follows: 125 samples from supermarkets, 126 samples from small shops, and 84 samples from open markets. Sampling of chicken carcasses was performed alternately between the north and south of the city. Each carcass was collected in its original bag and transported to the laboratory in an ice-box within the next 2 h. At the laboratory, 25 g of breast skin of every carcass were aseptically collected for bacteriological analysis.

Human stool samples: 302 samples were evenly collected in two health care centers located in the urban periphery of Quito (Guamani health care center at the south and Calderon health care center at the north) from patients with two or more episodes of diarrhea or vomiting in the last 24 h. Human stool samples were transported to the laboratory in an ice-box within the next 2 h. Approximately 25 g of feces were collected for bacteriological analysis.

According to national legislation, ethics approval was not required for poultry farms and chicken carcasses sampling since no animals were sacrificed during this study. For the human component, the project was approved by the bioethics committee from the National Institute of Public Health “Leopoldo Izquieta Pérez” (Protocol ID:CEISH-INSPI-005). The participants were informed about the objective of the study and all volunteers provided a written consent. All personal information was anonymized.

### Isolation and Identification of *Salmonella enterica*

*Salmonella* isolation was performed by a method based in the ISO 6579-1:2007 protocol. Briefly, 225 mL of Buffered Peptone Water (BPW; Difco, BD, Sparks, MD) was added to every sample, homogenized by hand for 1 min and incubated at 37°C for 20 h. Then, 100 μL of each enrichment was inoculated onto Modified Semi-solid Rappaport-Vassiliadis agar (MSRV; Oxoid, Basingstoke, UK) in three equidistant points and incubated at 42°C for 24 h. Afterwards, plates were examined for the presence of a white halo around of at least one inoculation point. A loopful taken from the edge of the white halo was streaked on a Xylose Lysine Deoxycholate agar (XLD, Difco) and incubated at 37°C for 24 h. After incubation, one suspect colony of *Salmonella* was biochemically confirmed by Triple Sugar Iron (TSI, Difco, BD), Iron Lysine (LIA, BBL, BD), Urea (BBL, BD), and Sulfur Indole Motility tests (SIM, BBL, BD). Isolated colonies were confirmed by PCR as previously described (16). The 95% confidence interval (CI_95%_) for the prevalence of *Salmonella* at each component was calculated.

### Antimicrobial Susceptibility Testing

All *Salmonella* isolates were examined by the Kirby-Bauer disk diffusion method with the following antibiotics: sulfamethoxazole + trimethoprim (25 μg), gentamicin (10 μg), ciprofloxacin (5 μg), cefotaxime (30 μg), tetracycline (30 μg), streptomycin (10 μg), chloramphenicol (30 μg), cefoxitin (30 μg), amikacin (30 μg), nitrofurantoin (300 μg), azithromycin (15 μg), fosfomycin (200 μg), ertapenem (10 μg), amoxicillin + clavulanic acid (30 μg). *E. coli* ATCC 25922 strain was used as quality control. Results and methods were interpreted according to CLSI 2019 criteria considering all intermediate phenotypes as resistant for further analysis (17).

### Detection of Extended Spectrum Beta-Lactamase (ESBL) Genes

*Salmonella* isolates that presented resistant phenotypes to beta-lactam antibiotics were further tested by PCR for the identification of ESBL genes. PCR conditions and primers were the ones described by Hasman et al. (18) for *bla*_*CTX*−*M*_, Olesen et. al (19) for *bla*_*TEM*_, Kruger et al. (20) for *bla*_*CMY*_ and Arlet et al. (21) for *bla*_*SHV*_. Sub-families of *bla*_*CTX*−*M*_ genes were identified with PCR protocols described by Carattoli et al. (22) for *bla*_*CTX*−*M*−1_, Jiang et al. (23) for *bla*_*CTX*−*M*−2_, Hopkins et al. (24) for *bla*_*CTX*−*M*−8_, Paauw et al. (25) for *bla*_*CTX*−*M*−9_ and Dierikx et al. (26) for *bla*_*CTX*−*M*−14_. Amplification products were confirmed by gel electrophoresis using a 2% agarose gel. All PCR products were purified and sequenced at Macrogen Inc (Seul-South Korea). Obtained sequences were aligned against reference sequences with the online tool ResFinder v3.2 (27).

### Whole Genome Sequencing

For whole genome sequencing (WGS), a selection of *Salmonella* isolates was made from the animal and food components. When selecting isolates from poultry farms the first positive sample of each farm was considered. For chicken carcasses, the first positive sample of every sampling week in each kind of market was selected. All non-*S*. Infantis isolates and all isolates from the human component were selected for WGS.

Genomic DNA was extracted using Invitrogen PureLink Genomic DNA Kit (Thermo Fisher Scientific, Walthman, MA, USA) following manufacturer's recommendations for Gram-negative bacterial cell lysates. DNA was quantified using Invitrogen Qubit 3.0 fluorometer (Thermo Fisher Scientific, Walthman, MA, USA), and sequenced with the Illumina NextSeq platform using Nextera XT Library Preparation Kit obtaining 150 × 2 bp paired-ends sequences (Illumina, San Diego, CA, USQ). Default parameters were used for all bioinformatic tools and programs unless otherwise specified. Reads were trimmed with Trimmomatic to remove ambiguous nucleotides and those with quality score values <20 (28). The programs Fastqc (29) and Multiqc (30) were used for quality assessment.

#### Serotype Identification

*Salmonella* serotypes were identified by PCR as described by Akiba et al. (16). Additionally, serotypes of isolates subjected to WGS were further confirmed by the analysis of their raw sequencing reads using the SeqSero pipeline (31).

#### MLST Analysis, Antimicrobial Resistance Genes, and Plasmid Detection

In order to identify MLST sequence types (ST), antimicrobial resistance genes and plasmid sequences, ARIBA (32) was used with PubMLST (33), ResFinder v3.2 (27), and PlasmidFinder 2.1 (34) databases, respectively. Phenotype resistance was compared with the presence of resistance genes found by WGS. Additionally, we performed a mapping against the megaplasmid p-F219 described by Vallejos-Sánchez et al. (35) using Burrows-Wheeler Aligner with BWA-MEM algorithm (36). BCFtools and vcfutils from SAMtools (37) were used to obtain the fastq files from SAM files, and the fasta sequences were transformed from fastq with Seqtk (38). The sequences were concatenated and a maximum likelihood phylogenetic tree was obtained with IQTREE2 software (39). As we obtained two well-defined clusters for *S*. Infantis, we annotated one representative of each plasmid cluster with Prokka (40) and performed an orthologous genes analysis (coverage of 90%, similarity on protein sequences of 80%) with Proteinortho5 (41). A manual comparison of all genes present in the plasmids was carried out. We performed the same analysis for non-Infantis isolates.

#### Megaplasmid Analysis

Two megaplasmids (pESI and p-F219) commonly associated to pathogenic and MDR strains of *S*. Infantis were analyzed in order to identify their relatedness. We used D-Genies (42) to obtain a dot plot of genome comparison, a genome alignment with progressiveMauve (43) in order to identify locally collinear blocks, and an ANI calculation (44) for computing average nucleotide identity in sequences shared by both plasmids.

#### Core Genome and Metadata Analysis

A Peruvian *S*. Infantis strain, FARPER-219 (35), and two Ecuadorian isolates (SRR4019589 and SRR4019602) analyzed by the US Centers for Disease Control and Prevention from two patients that developed salmonellosis after traveling to Ecuador (10) were added as references in the phylogenetic analysis of all *S*. Infantis isolates. From trimmed reads, Spades (45) was used to generate assemblies. Later, genome annotation was performed with Prokka (40). An orthologous genes analysis with the same conditions as for plasmid detection was performed with Proteinortho5 (41). The strict core genes, those present in all the isolates, were extracted with the Proteinortho tool: grab_proteins.pl. Mafft (46) and an in-house script were used for multiple alignment of every gene and subsequent concatenation in a single multiple alignment, respectively. The phylogenetic tree from the core genome alignment was obtained using IQTREE2 (39) with 1,000 bootstrap replicates. The metadata for sample origin, phenotypic antibiotic resistance patterns, and plasmid *in silico* detection was added to the final tree with iTol tools (47).

## Results

### *Salmonella* Prevalence and Serotype Identification

*Salmonella* was present in 41.4% (55/133; CI_95%_:33–49.7), 55.5% (186/335; CI_95%_:50.2–60.8), and 1.98% (6/302; CI_95%_:0.4–3.6) in poultry farms, chicken carcasses and human stool samples, respectively. *S*. Infantis accounted for 98.2% (*n* = 54) of isolates from poultry farms, 97.8% (*n* = 182) of isolates from chicken carcasses, and one half (*n* = 3) of human sample isolates. Additionally, one isolate was typed as *S*. Enteritidis in broiler flocks; at the retail level one and three isolates were typed as *S*. Typhimurium and *S*. Enteritidis, respectively, while in the human stool samples two isolates were typed as *S*. Enteritidis and one isolate corresponded to monophasic *S*. Typhimurium 4,[5],12:i:- (Supplementary Table 1).

### Antimicrobial Resistance

For *S*. Infantis isolates, antimicrobial resistance rates to nitrofurantoin, tetracycline, sulfamethoxazole + trimethoprim, streptomycin, gentamicin, cefotaxime, ciprofloxacin and chloramphenicol ranged from 64.8 to 100%. On the other hand, fosfomycin and azithromycin resistance rates were lower, ranging from 0 to 42.6%. Only one isolate from a stool sample presented phenotypic resistance to amikacin while none of the *Salmonella* isolates in this study was resistant to ertapenem (Table 1).

**Table 1:** Number of *S*. Infantis isolates resistant to each tested antimicrobial.

**Antimicrobial**	**Number (%) of resistant isolates**		
	**Poultry farms (farm)**	**Chicken carcasses (food)**	**Stool samples (human)**
Nitrofurantoin	54 (100)	180 (99.4)	2 (66.7)
Tetracycline	54 (100)	176 (97.2)	3 (100)
Sulfamethoxazole + trimethoprim	44 (81.5)	158 (87.3)	1 (33.3)
Streptomycin	46 (85.2)	154 (85.1)	3 (100)
Gentamicin	45 (83.3)	155 (85.6)	2 (66.7)
Cefotaxime	51 (94.4)	150 (82.9)	1 (33.3)
Chloramphenicol	45 (83.3)	149 (82.3)	2 (66.7)
Ciprofloxacin	35 (64.8)	116 (64.1)	1 (33.3)
Fosfomycin	23 (42.6)	68 (37.6)	1 (33.3)
Azithromycin	10 (18.5)	31 (17.1)	0 (0)
Cefoxitin	7 (13)	11 (6.1)	0 (0)
Amoxicillin + clavulanic acid	7 (13)	7 (3.9)	0 (0)
Amikacin	0 (0)	0 (0)	1 (33.3)
Ertapenem	0 (0)	0 (0)	0 (0)

Considering antimicrobial classes that were tested, *S*. Infantis isolates presented 43 antimicrobial resistance patterns. With the exception of one isolate from a stool sample, all isolates showed multidrug-resistant phenotypes. Importantly, 87 and 82% of isolates from poultry farms and chicken carcasses, respectively, presented resistance from 6 up to 9 classes of antimicrobials (Supplementary Table 2). One *S*. Infantis isolated from chicken carcasses could not be recuperated for this analysis.

*Salmonella* serotypes other than *S*. Infantis also presented multiresistant patterns, except for 3 *S*. Enteritidis isolates that were only resistant to one group of antimicrobials. For this set of isolates, every resistant pattern included isolates belonging to only one serotype (Table 2).

**Table 2:** Antimicrobial resistance patterns of *S*. Enteritidis, *S*. Typhimurium, and monophasic *S*. Typhimurium 4,[5],12:i:-.

**Resistant pattern**	**No. Antimicrobial clases**	***S*. Enteritidis**	***S*. Typhimurium**	**Monophasic *S*. Typhimurium 4,[5],12:i:-**
SAQBTFN	7		1	
SAQTFNM	7	1		
SABTFNP	7	1		
SABTFN	6			1
QFM	3	1		
N	1	3^*^		

* *1 isolate obtained from poultry farms and 2 isolates from human stool samples*.

*Sulfonamide (S), aminoglycosides (A), quinolones (Q), Beta-lactams (B), tetracyclines (T), phenicol (F), nitrofuran (N), macrolides (M), Fosfomycin (P)*.

One isolate of *S*. Enteritidis, one of *S*. Typhimurium and one of Monophasic *S*. Typhimurium 4,[5],12:i:-; and 205 isolates of *S*. Infantis were identified as resistant to beta-lactam antibiotics.

Six *S*. Infantis isolates from chicken carcasses and one from poultry farms did not present any of the investigated ESBL genes. All other *S*. Infantis and one *S*. Enteritidis isolated from a carcass presented the *bla*_CTX−M−65_ gene.

### Genomic Analysis

For WGS analysis, 144 isolates (40 from the animal component, 98 from the food component and six from the human component) were selected. Raw sequence data is available under bioproject PRJEB37560. The sequences from three samples were not enough to perform genomic analysis. The obtained average number of reads per strain was 1,356,678 (range 247,022–14,106,025) and after the quality control steps, the average number was 1,266,242 (range 228,263–13,094,594) (Supplementary File 1). Average Phred Score was Q34.

MLST typing showed that all *S*. Infantis isolates (*n* = 137) belonged to ST32. The five *S*. Enteritidis isolates belonged to ST11. Additionally, the single isolates of *S*. Typhimurium and monophasic *S*. Typhimurium 4,[5],12:i:- belonged to ST19 and ST2379, respectively (Supplementary Table 1).

**Figure 1:**
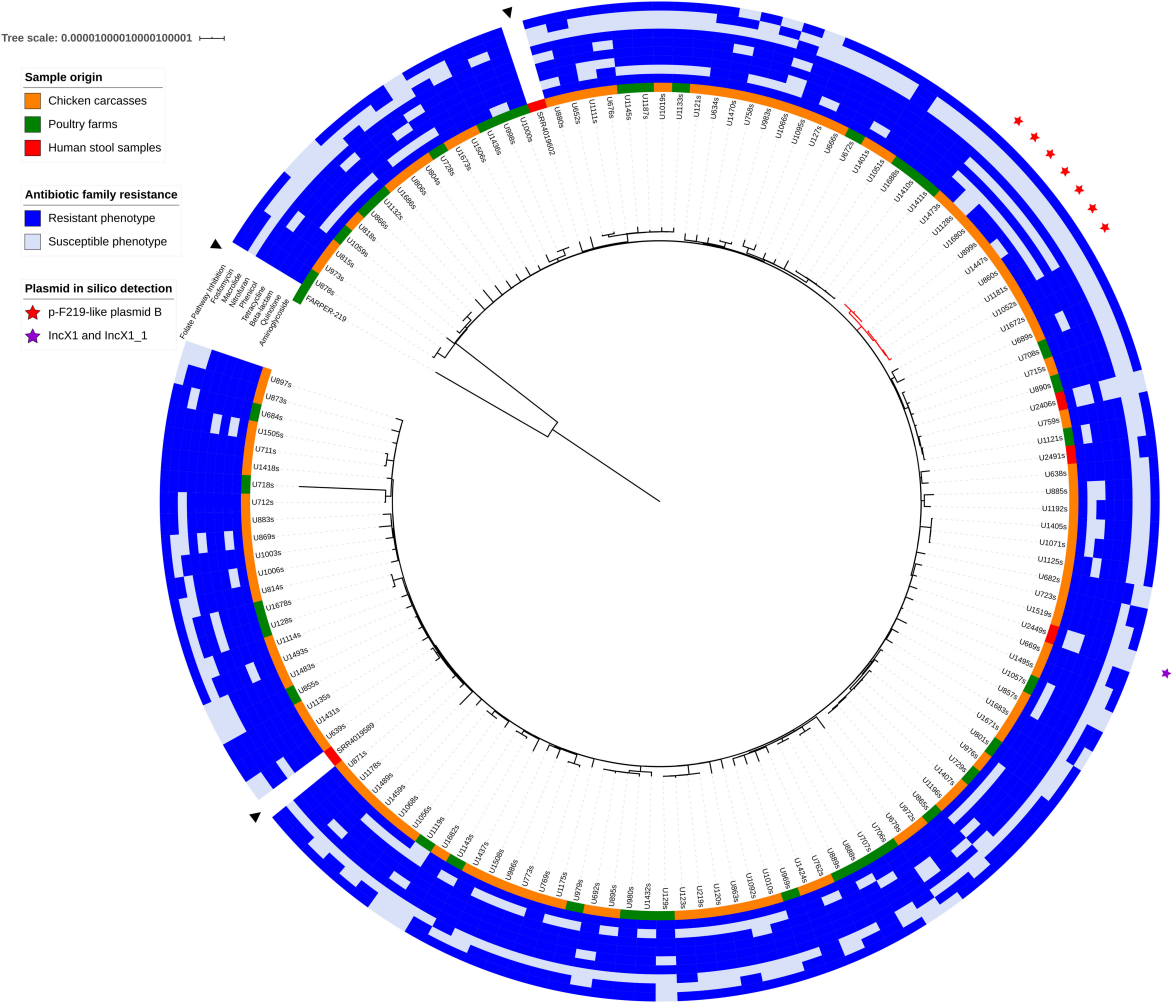
Maximum likelihood phylogenetic tree of core genome alignment of all 137 *S*. Infantis isolates based on 3,552 genes. Two *S*. Infantis genomes from Ecuador detected in USA (SRR4019589, SRR4019602) and a Peruvian strain (FARPER-219) were included in the analysis and are indicated with a black triangle. The origin of each sample is colored in red for human stool samples, in green for poultry farms isolates and in orange for chicken carcasses strains. The phenotypic resistance for nine antibiotic families is marked with a blue box. Strains with p-F219-like plasmid B are marked with a red star. The rest of the samples harbor the p-F219-like plasmid A. The purple star indicates the presence of IncX1 and IncX1_1 plasmids in one of the strains. Digital version of the phylogenetic tree is available with iTOL login LMejia at https://itol.embl.de/shared_projects.cgi.

The strict core genome of all *S*. Infantis included in the analysis corresponded to 3,552 genes and spanned 3,161,448 bp, 1,414 of which were variable (SNPs). The alignment of the concatenated genes present in this core was used to obtain a maximum-likelihood tree using FARPER 219 as outgroup (Figure 1). This strain was chosen because it was isolated in Peru, a neighbor country to Ecuador. Despite its inclusion in ST32, FARPER-219 presented genetic divergence with the Ecuadorian strains. The two *Salmonella* genomes from the USA (SRR4019589, SRR4019602) grouped indistinctive with some of the genomes of this study. Notably, the analyzed strains did not group according to their sampling origin or their phenotypic resistance patterns.

Genes of antimicrobial resistance were also confirmed with WGS data (Supplementary File 2). For most of the antimicrobial classes (folate pathway inhibition, aminoglycoside, beta-lactams, tetracyclines, fosfomycin, and phenicol) correspondence rates were higher than 80%. However, no genes responsible for the phenotypic resistance to quinolones, nitrofurans, and macrolides were found in sequenced isolates (Table 3).

**Table 3:** Comparison of phenotypic AMR with AMR genes obtained from WGS data.

**Antibiotic family**	**Phenotype (%)^a^**	**Phenotype + AMR gene^b^ (%)**	**No phenotype + AMR gene^c^ (%)**
Folate pathway inhibition	84.56	92.17	7.83
Aminoglycoside	97.06	100	3.03
Quinolone	58.09	1.27	0
Beta-lactams	84.56	94.87	4.27
Tetracycline	97.06	99.24	0.76
Phenicol	80.88	80.91	6.36
Nitrofuran	98.53	0	0
Macrolide	17.65	0	0
Fosfomycin	37.5	90.2	41.18

a *Rate of isolates with phenotypic resistance*.

b *Rate of isolates with phenotypic resistance that presented a resistance gene by WGS analysis*.

c *Rate of isolates without phenotypic resistance that presented a resistance gene by WGS analysis*.

Regarding virulence genes, *sifA, sseL, pipB, sopD2*, and *srlP* (part of SPI 2) were found in the core genome of all *S*. Infantis strains. The *lpf* operon that encodes the long polar fimbrae (LPF), the *fim* gene cluster that encodes type 1 fimbriae, and the *csg* operons that encodes the Tafi fimbriae (Thin aggregative fimbriae) were also present in these genomes.

The presence of plasmids was confirmed *in silico* by PlasmidFinder. Only one *S*. Infantis isolate (U2449s) presented one plasmid determinant (IncX1 and IncX1_1) despite the multidrug resistance patterns found in our isolates (Figure 1 and Supplementary File 2). To further analyze the low incidence of plasmids, we mapped the raw reads from our samples against the megaplasmid p-F219 described by Vallejos-Sánchez et al. (35). We found two p-F219-like plasmids. The first one, denoted as plasmid A, was present in most of the strains. This plasmid contained 338 genes shared with the p-F219 megaplasmid. The second one, denoted as plasmid B, was present in the remaining seven strains. Plasmid B lacked 72 of the genes present in plasmid A and presented six exclusive genes (Supplementary File 3). The strains that presented plasmid B belonged to a monophyletic clade (denoted with a red star in Figure 1). These strains also share susceptibility to fosfomycins, macrolides, phenicols, and beta-lactams. *Salmonella* strains harboring plasmid B were isolated from chicken carcasses sampled during different weeks of the year, different parts of the city and different types of retail stores (data not shown).

New hypothetical proteins (*n* = 147) were found in both plasmids; 43 exclusively found in plasmid A and five in plasmid B (Supplementary File 3).

The comparison of the p-F219 plasmid with another megaplasmid, commonly found in pathogenic *S*. Infantis strains, pESI plasmid, showed that they share more than 79% of their sequences (>75% of identity) (Supplementary Figure 1). Besides, we noticed a large genomic inversion in plasmid p-F219 when compared with the pESI plasmid that is also observable in progressiveMauve genome alignment (Supplementary Figure 2). From the ANI calculation, 99.41% of identity was found in the orthologous genes present in both samples.

*In silico* plasmid detection in non-Infantis isolates showed the presence of two plasmids in all *S*. Enteritidis strains, while monophasic *S*. Typhimurium 4,[5],12:i:- and *S*. Typhimurium presented 1 and 5 plasmids, respectively (Supplementary Table 1).

## Discussion

In the last decades, there has been a clear rise in the prevalence of multidrug resistant *Salmonella enterica* worldwide, especially of serovar Infantis (11, 48–50).

To better explain the epidemiology of *Salmonella* in Ecuador, we studied *S*. *enterica* isolated from poultry farms, chicken carcasses and human stool samples in Quito. The prevalence of *Salmonella* was high in poultry farms and chicken carcasses, similarly to other studies in the region (6–8, 51). Although this research did not look for *Salmonella* in earlier stages of the broiler production chain, previous studies in Ecuador have reported the importance of compound feed, 1-day-old chicks and broiler pens in the *Salmonella* exposure of broilers (7, 52). These results highlight the necessity to improve broilers production systems in Ecuador toward a better control of *Salmonella* in the food chain.

In this study, it was also seen that samples from different kinds of markets delivered similar rates of *Salmonella* isolates, denoting that the geographical distribution of retailers does not influence the presence of *Salmonella* in carcasses (Supplementary Table 3). On the other hand, all human isolates originated in the southern health care center. However, it must to be considered that the low *Salmonella* prevalence in human samples could be influenced by the fact that other pathogens might be the main causes of diarrhea. This fact has been studied in Ecuador were other viruses, parasites and bacteria are the main cause of gastroenteritis cases (53–56). Additionally, the state of health carriers should be considered when accessing the real prevalence of *Salmonella* in humans (57). These circumstances represent limitations of this study and should be considered in future research.

The predominance of *S*. Infantis in this study is in accordance with other reports in the world that show that this serotype is becoming an emergent pathogen. For example, in Europe, *S*. Infantis has been reported to be one of the most common serovars in poultry and in humans (58). The same tendency has been reported in the neighboring country of Peru, where *S*. Infantis is the most prevalent serotype in broilers (6). However, a wider variety of serotypes has been reported in other Latin American countries (51, 59, 60). This could be explained by the fact that the poultry industry of Peru and Ecuador have close commercial interactions which could determine a common epidemiology of this pathogen. Nevertheless, a recent publication from Chile reported that 24% of broiler meat samples (*n* = 361) were positive to the isolation of *S*. Infantis (61). This data highlights the necessity of more research in the field to better understand the epidemiology of *Salmonella* in the region.

Several studies have shown that control programs of targeted *Salmonella* serotypes could have favored the occurrence of other serotypes (2, 62). This kind of shifts might explain to some extent the low presence of *S*. Enteritidis and Typhimurium among our samples but further research is needed to prove this hypothesis.

Most *Salmonella* isolates in this study presented multidrug resistance (MDR) phenotypes. This issue is especially evident in *S*. Infantis, as already been seen in Ecuador (7, 8) and other countries of the region (63–65). Although in a lower extent, this feature has also been reported in Europe where high rates of MDR (up to 85%) are reported in *S*. Infantis isolated from poultry (66). The high levels of resistance in *Salmonella* isolates in Latin America could be related to the intensive use of antimicrobials in poultry production as prophylactics, therapeutics, and growth promotors (67, 68).

*S*. Infantis isolates belonged to ST32, that is among those more frequently identified within this serovar (33, 69). All Ecuadorian *S*. Infantis isolates showed a high genomic similarity with an apparently common origin. However, in order to verify that all in fact share a common ancestor, a larger analysis including isolates from countries around the world is necessary for identifying the origin of this serovar in Ecuador. The close similarity found between isolates from farms, animals and humans show that this pathogen may be responsible for human infections through the food chain.

We did not observe any clear clustering of *S*. Infantis isolates and antibiotic phenotypic resistance patterns, what makes sense since the phylogenetic tree was obtained from the core genome and most of the antimicrobial resistance determinants are expected to be part of the accessory genome.

The resistance genes analysis carried out here could not successfully explain resistance to macrolides, nitrofurans, and quinolones in our isolates. In fact, phenotypic patterns and genetic detection correlation differences have been described previously (70–72), but point mutations not considered in this study may explain these phenotypes. Additional studies are still needed in order to identify the genes or mutations responsible for antimicrobial resistance in *S*. Infantis.

We found two p-F219-like plasmids (named A and B) present in all the analyzed isolates. Our samples share a significant number of genes with the p-F219 plasmid. The similarity between both p-F219-like plasmids requires a recent common ancestor from which they may have evolved. These plasmids may provide fitness and pathogenic advantages to the strains since they contain several genes for antimicrobial resistance, fimbriae, transposases, and environmental stress tolerance (Supplementary File 3). Other *S*. Infantis isolates around the world have been shown to also harbor pESI-related megaplasmids, that appear to be the difference between non-pathogenic and pathogenic isolates since it also provides antimicrobial resistance, oxidative stress tolerance, pathogenicity traits, and mercury environmental tolerance (69, 70, 73, 74). Besides, pESI has shown more pathogenicity and increased intestinal inflammation in experimental mice infections when compared to the plasmid-free isolates (73).

Both plasmids (p-F219 and pESI) share regions of very close similarity with an extensive genomic inversion. Genes present in both plasmids share more than 99% average nucleotide identity suggesting that p-F219 is actually a pESI-like plasmid and the variants found in our samples may also be cataloged as such. A genomic comparison between *S*. Infantis strains showed that large plasmids from multiple isolates actually share a pESI backbone with some genetic plasticity to add different mobile genetic elements due to insertion sequences (75). As pESI-like plasmids confer a MDR phenotype but also several virulence factors and tolerance to environmental stress, their acquisition may have been involved for making *S*. Infantis a successful emerging pathogen worldwide.

The negative results from the unmapped reads from *S*. Enteritidis and *S*. Typhimurium isolates against the p-F219 plasmid suggest that this plasmid may not be widespread among other *Salmonella* serovars, but more isolates need to be analyzed to confirm this hypothesis.

Other virulence genes in *Salmonella* are essential for pathogenicity and infection. We looked for some of the more relevant virulence and invasion determinants in the core genome of *S*. Infantis as determined by ProteinOrtho. Some genes that are usually identified as part of the *Salmonella* pathogenicity island 2 (SPI-2) were found: *sifA, sseL, pipB, sopD2, srlP* (75). The long polar fimbrae (LfP), encoded by the *lpf* operon, was found as putative proteins in all isolates; it is believed that this protein is involved in adhesion and growth on the small intestine mucosa (76), evasion of the host immune system (77). Moreover, variations in these genes may also impact the host range of the bacteria (78). We also found the *fim* gene cluster that encodes type 1 fimbriae and it is involved in the initiation of biofilm formation (79). Another determinant found in our strains was the *csg* operon that encodes the Tafi fimbriae (Thin aggregative fimbriae). Tafi is responsible of adhesive activities and biofilm formation (76). The presence of these genes in *S*. Infantis may suggest a pathogenic character of this serotype that, together with the multidrug resistant profiles, represent a potential public health concern.

To the best of our knowledge this is the first report based on *Salmonella enterica* in Ecuador that uses phenotypic and WGS information to analyze the relatedness of strains isolated from poultry, food and human samples. Isolates from this study show multidrug resistance patterns highlighting the importance of a reduced and better usage of antimicrobials in intensive poultry farms settings. Presence of related megaplasmids together with the core genome high similarity may suggest the dissemination of *S*. Infantis through the food chain to humans. The data presented here has shown the importance of *Salmonella enterica* serovar Infantis as a foodborne pathogen in Ecuador and provide critical information about its clonality and circulating strains.

## Data Availability Statement

Sequence data are available under bioproject PRJEB37560.

## Ethics Statement

According to national legislation, ethics approval was not required for poultry farms and chicken carcasses sampling since no animals were sacrificed during this study. For the human component, the project was approved by the bioethics committee from the National Institute of Public Health Leopoldo Izquieta Pérez (Protocol ID was CEISH-INSPI-005). The participants were informed about the study's objective and all volunteers provided a written consent. All personal information was anonymized.

## Author Contributions

JM, JW, FV, and CV-B conception and design of the study. JLM, CS, FV, and RB performed laboratory analysis. JLM and CV-B performed the statistical analysis. JLM, LM, and CV-B organized the databases. LM and FG-C performed the bioinformatic and genomic analysis. CV-B and LM wrote the first draft of the manuscript. LM, SZ, FG-C, and CV-B contributed to the manuscript revision. All authors contributed to the article and approved the submitted version.

## Conflict of Interest

The authors declare that the research was conducted in the absence of any commercial or financial relationships that could be construed as a potential conflict of interest.
