# Supplementary material for: Genomic Epidemiology of Salmonella Infantis in Ecuador: From Poultry Farms to Human Infections
Source: Front Vet Sci. 2020 Sep 29;7:547891. doi: 10.3389/fvets.2020.547891 (PMC7550756; doi:10.3389/fvets.2020.547891)
Supplement: Supplementary file 1 [file Table_1.docx]

**Supplementary Table 1.** Sequence Type and Plasmid determinants present in non-*S*. Infantis isolates. Detection was done in silico with PubMLST and PlasmidFinder**.**

| **Sample ID** | **Serovar** | **ST** | **Col156** | **Col8282** | **ColRNAI** | **IncFIB** | **IncFII_S** | **IncQ1** |
| --- | --- | --- | --- | --- | --- | --- | --- | --- |
| U875s | Enteritidis | 11 | 0 | 0 | 0 | 1 | 1 | 0 |
| U114s | Enteritidis | 11 | 0 | 0 | 0 | 1 | 1 | 0 |
| U1193s | Enteritidis | 11 | 0 | 0 | 0 | 1 | 1 | 0 |
| U2506s | Enteritidis | 11 | 0 | 0 | 0 | 1 | 1 | 0 |
| U2526s | Enteritidis | 11 | 0 | 0 | 0 | 1 | 1 | 0 |
| U2451s | Monophasic Typhimurium 4,[5],12:i:-. | 2379 | 1 | 0 | 0 | 0 | 0 | 0 |
| U113s | Typhimurium | 19 | 0 | 1 | 1 | 1 | 1 | 1 |
