# Supplementary material for: Genomic Epidemiology of Salmonella Infantis in Ecuador: From Poultry Farms to Human Infections
Source: Front Vet Sci. 2020 Sep 29;7:547891. doi: 10.3389/fvets.2020.547891 (PMC7550756; doi:10.3389/fvets.2020.547891)
Supplement: Supplementary file 2 [file Table_2.docx]

**Supplementary Table 2.** Antibiotic resistance patterns of *S*. Infantis.  Sulfonamide (S), Aminoglycosides (A), Quinolones (Q), Beta-lactams (B), Tetracyclines (T), Phenicol (F), Nitrofuran (N), Macrolides (M), Fosfomycin (P).

| **Resistant  pattern** | **No.  Antimicrobial**  **classes** | **Sample  origin** | | | |
| --- | --- | --- | --- | --- | --- |
|  |  | **Poultry farms (farm)** | **Chicken carcasses (food)** | **Stool samples**  **(human)** | **Total** |
| SAQBTFNMP | 9 | 3 | 10 |  | 13 |
| SAQBTFNP | 8 | 10 | 26 | 1 | 37 |
| SAQBTFNM | 8 | 2 | 10 |  | 12 |
| SABTFNMP | 8 | 1 | 1 |  | 2 |
| AQBTFNMP | 8 |  | 1 |  | 1 |
| SAQBTFN | 7 | 9 | 28 |  | 37 |
| SAQTFNM | 7 |  | 1 |  | 1 |
| SABTFNP | 7 | 6 | 19 |  | 25 |
| SABTFNM | 7 |  | 1 |  | 1 |
| SAQBTNM | 7 | 2 |  |  | 2 |
| AQBTFNM | 7 | 1 | 2 |  | 3 |
| SAQBTNP | 7 |  | 1 |  | 1 |
| SABTNMP | 7 | 1 |  |  | 1 |
| AQBTFNP | 7 | 1 | 3 |  | 4 |
| SQBTFNP | 7 | 1 |  |  | 1 |
| SABFNMP | 7 |  | 1 |  | 1 |
| SABTFN | 6 | 5 | 22 |  | 27 |
| AQBTNM | 6 |  | 2 |  | 2 |
| SAQTFN | 6 | 2 | 6 |  | 8 |
| SAQBTN | 6 | 1 | 4 |  | 5 |
| SABFNP | 6 |  | 1 |  | 1 |
| AQBTFN | 6 | 2 | 6 |  | 8 |
| ABTFNP | 6 |  | 1 |  | 1 |
| AQBFNP | 6 |  | 1 |  | 1 |
| AQBTNP | 6 |  | 1 |  | 1 |
| SAQTNM | 6 |  | 1 |  | 1 |
| AQBTN | 5 | 1 | 1 |  | 2 |
| SAQTN | 5 |  | 8 |  | 8 |
| AQBNP | 5 |  | 1 |  | 1 |
| ABTFN | 5 | 2 | 2 |  | 4 |
| SABTN | 5 | 1 | 5 |  | 6 |
| SATFN | 5 |  | 6 |  | 6 |
| SATNM | 5 |  | 1 |  | 1 |
| SABFP | 5 |  | 1 |  | 1 |
| AQTN | 4 |  | 1 |  | 1 |
| ABTN | 4 | 1 |  |  | 1 |
| SATN | 4 |  | 1 |  | 1 |
| SQTN | 4 |  | 2 |  | 2 |
| ATFN | 4 |  |  | 1 | 1 |
| BTN | 3 | 2 | 1 |  | 3 |
| STN | 3 |  | 2 |  | 2 |
| AT | 2 |  |  | 1 | 1 |
| Total |  | 54 | 181 | 3 | 239 |
