# Supplementary material for: Genomic Epidemiology of Salmonella Infantis in Ecuador: From Poultry Farms to Human Infections
Source: Front Vet Sci. 2020 Sep 29;7:547891. doi: 10.3389/fvets.2020.547891 (PMC7550756; doi:10.3389/fvets.2020.547891)
Supplement: Supplementary file 3 [file Table_3.docx]

**Supplementary Table 3.** *Salmonella* isolates in retailers and health care centers located in two different zones of Quito

| **Origin of sample** | ***Salmonella* isolates (Number of samples)** | | |
| --- | --- | --- | --- |
|  | **North**  **Zone** | **South**  **Zone** | **Total** |
| Open markets | 25 (42) | 23 (42) | 48 (84) |
| Supermarkets | 31 (61) | 33 (64) | 64 (125) |
| Small shops | 38 (63) | 35 (63) | 73 (126) |
| Total | 94 (166) | 92 (169) | 186 (335) |
| Health Care Centers | 0 (151) | 6(151) | 6 (302) |
