# Supplementary material for: Genomic Epidemiology of Salmonella Infantis in Ecuador: From Poultry Farms to Human Infections
Source: Front Vet Sci. 2020 Sep 29;7:547891. doi: 10.3389/fvets.2020.547891 (PMC7550756; doi:10.3389/fvets.2020.547891)
Supplement: Supplementary file 6 [file Data_Sheet_3.docx]

**Supplementary file 3**. List of genes present in the p-F219-like plasmids found in all samples.

**Proteins present in both plasmids**

2,3-dihydroxybenzoate-AMP ligase

Adenosine monophosphate-protein transferase SoFic

Anaerobic nitric oxide reductase transcription regulator NorR

Antirestriction protein KlcA

Antitoxin CcdA

Arginine/agmatine antiporter

ATP-dependent RNA helicase DbpA

Biodegradative arginine decarboxylase

Chaperone protein FaeE

Cyclic di-GMP phosphodiesterase PdeL

D-alanine--poly(phosphoribitol) ligase subunit 1

Dihydropteroate synthase

DNA adenine methyltransferase YhdJ

Endoribonuclease PemK

Inner membrane transport protein YhjV

IS110 family transposase ISEc21

IS110 family transposase ISSfl8

IS200/IS605 family transposase IS609

IS21 family transposase IS1326

IS21 family transposase ISEc10

IS256 family transposase IS285

IS256 family transposase ISEc39 (n=2)

IS256 family transposase ISSod4

IS3 family transposase ISEam1

IS3 family transposase ISIba2

IS3 family transposase ISKpn40 (n=2)

IS3 family transposase ISPa74

IS3 family transposase ISYpe1

IS4 family transposase ISSfl1 (n=2)

IS481 family transposase ISErsp1

IS630 family transposase ISEc40

ISL3 family transposase ISStma11

ISNCY family transposase ISRor2 (n=3)

K88 fimbrial protein AC

L-methionine gamma-lyase

Lactococcin-G-processing and transport ATP-binding protein LagD

Leader peptidase PppA

Lipoprotein signal peptidase

Low affinity potassium transport system protein kup

Major fimbrial subunit SMF-1

Mercuric reductase

Multidrug transporter EmrE

N-acetylmuramoyl-L-alanine amidase AmiD

Nucleoid occlusion protein

Outer membrane usher protein HtrE

Pesticin receptor

Phthiocerol synthesis polyketide synthase type I PpsE

Plasmid-derived single-stranded DNA-binding protein

Protein AmpG

Protein PsiB

Protein SopB

Protein UmuD

Protein YgiW

putative fimbrial chaperone YadV

putative protein YggR

putative protein YjiK

putative signaling protein

putative transport protein HsrA

RepFIB replication protein A

S6 family transposase IS26 (n=6)

Salicylate synthase

Streptomycin 3''-adenylyltransferase

Succinate-acetate/proton symporter SatP

Surfactin synthase thioesterase subunit

Tellurite resistance protein TehA

Tetracycline repressor protein class A from transposon 1721

Tetracycline resistance protein, class C

Tn3 family transposase TnAs1

Toxin CcdB

Transcription antitermination protein RfaH (n=2)

tRNA(fMet)-specific endonuclease VapC

Tyrosine recombinase XerC (n=6)

Ubiquinone/menaquinone biosynthesis C-methyltransferase UbiE

Vitamin B12 import ATP-binding protein BtuD (n=2)

Hypothetical proteins (174 different ORFs)

**Proteins exclusive to plasmid A**

Antitoxin PemI

Arsenical pump-driving ATPase

Arsenical resistance operon trans-acting repressor ArsD

Beta-lactamase Toho-1

Bicyclomycin resistance protein

ECF RNA polymerase sigma factor SigL

F1 capsule-anchoring protein

Glutathione transferase FosA

HTH-type transcriptional regulator HdfR

Hygromycin-B 4-O-kinase

IS200/IS605 family transposase ISShwo2

IS21 family transposase ISEc57

IS5 family transposase IS903

IS6 family transposase ISRle7

Lipopolysaccharide core heptose(II)-phosphate phosphatase

Mercuric transport protein MerT

Mercuric transport protein periplasmic component

NADPH-dependent FMN reductase ArsH

Plasmid segregation protein ParM

Protein PndA

Protein UmuC

putative outer membrane usher protein LpfC

Signal recognition particle 54 kDa protein

SPBc2 prophage-derived aminoglycoside N(3')-acetyltransferase-like protein YokD

Tn3 family transposase

Transcriptional repressor PifC

Tyrosine recombinase XerD

Vitamin B12 transporter BtuB

Hypothetical proteins (43 different ORFs)

**Proteins exclusive to plasmid B**

IS3 family transposase ISYps8

Hypothetical proteins (5 different ORFs)
