# Supplementary material for: Genomic Epidemiology of Salmonella Infantis in Ecuador: From Poultry Farms to Human Infections
Source: Front Vet Sci. 2020 Sep 29;7:547891. doi: 10.3389/fvets.2020.547891 (PMC7550756; doi:10.3389/fvets.2020.547891)
Supplement: Supplementary file 7 [file Image_1.pdf]

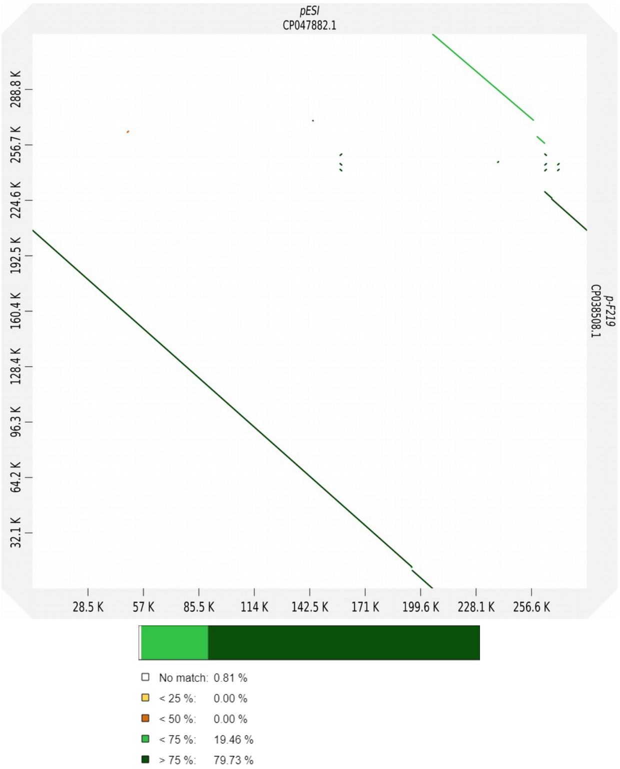

Supplementary Figure 1. Dot Plot of genome comparison of pESI and p-F219 plasmids obtained with D-Genies aligner.
