# Supplementary material for: Genomic Epidemiology of Salmonella Infantis in Ecuador: From Poultry Farms to Human Infections
Source: Front Vet Sci. 2020 Sep 29;7:547891. doi: 10.3389/fvets.2020.547891 (PMC7550756; doi:10.3389/fvets.2020.547891)
Supplement: Supplementary file 8 [file Image_2.pdf]

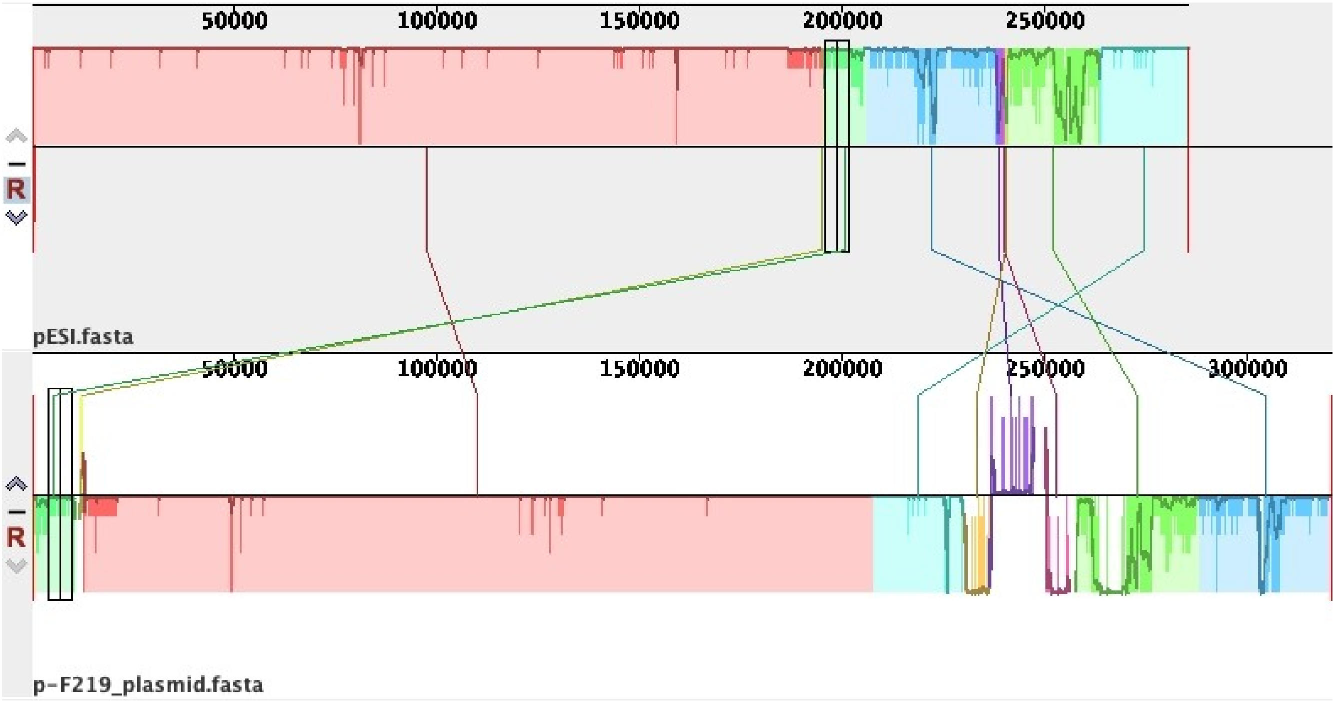

Supplementary figure 2. Locally collinear blocks (LCBs) found in the genome alignment of pESI and p-F219 plasmids obtained with progressiveMauve.

LCBs are colored in order to identify the conserved segments that appear to be internally free from genome rearrangements and presumably homologous. The LCBs that appear below the center line indicate regions with inverse orientation.
